# Intraluminal Insertion of 9-0 Nylon for Postoperative Choroidal Detachment After Preserflo MicroShunt Implantation: A Case Report

**DOI:** 10.7759/cureus.74560

**Published:** 2024-11-27

**Authors:** Yuta Kitamura, Shunsuke Ikema, Tomoaki Tatsumi, Takayuki Baba

**Affiliations:** 1 Department of Ophthalmology and Visual Science, Chiba University Graduate School of Medicine, Chiba, JPN

**Keywords:** case report, choroidal detachment, glaucoma, intraocular pressure, preserflo microshunt

## Abstract

Preserflo MicroShunt (PMS) implantation is a minimally invasive surgical procedure for treating glaucoma. Postoperative hypotony, a common complication of PMS implantation, can be prevented and treated with 10-0 nylon insertion. In this report, we present a case of postoperative hypotony following PMS implantation that was treated with intraluminal insertion of 9-0 nylon. A 78-year-old woman with immunoglobulin G4 (IgG4)-related disease and nephrotic syndrome was being treated with oral steroids for more than 15 years. She developed steroid-induced glaucoma in both eyes. At presentation, her intraocular pressures (IOPs) were markedly elevated to nearly 40 mmHg, which caused severe visual dysfunction. We performed PMS implantation in both eyes. The postoperative IOP was maintained at 7-10 mmHg; however, the patient experienced severe choroidal detachment (CD) extending to the posterior pole and shallowing of the anterior chamber in both eyes. After inserting 9-0 nylon into the lumen of the PMS in both eyes, the IOP increased to 25-30 mmHg. Additionally, the CD completely resolved within a month of the procedure. The administration of glaucoma eye drops resulted in a sustained IOP of 15 mmHg. When the 9-0 nylon was removed in the left eye only, the IOP decreased to 8 mmHg, which caused the recurrence and persistence of CD for six months postoperatively. Insertion of 9-0 nylon into the lumen of a PMS may be effective for treating postoperative hypotony; however, future studies are warranted to evaluate the optimal timing for stent removal.

## Introduction

Preserflo MicroShunt (PMS) implantation is a minimally invasive filtration surgery alternative to conventional trabeculectomy. An increasing number of PMS implantation surgeries are being performed both in Japan and in other countries. The intraocular pressure (IOP)-lowering efficacy and safety of this procedure are comparable with those of trabeculectomy [1-3]. A recent meta-analysis revealed that trabeculectomy is more effective in patients with uncontrolled glaucoma for up to two years [4]. Postoperative complications include elevated IOP resulting from tube occlusion, a shallow anterior chamber, choroidal detachment (CD), and hypotony maculopathy [5-8]. Recent reports have discussed the efficacy of nylon stent placement for preventing and treating decreased postoperative IOP complications [9-11]. Although clinicians have reported inserting 10-0 nylon, the effect of 9-0 nylon implantation on IOP elevation remains uninvestigated. In addition, consensus regarding the optimal timing for stent removal following nylon insertion is lacking. In this report, we present the case of a patient with postoperative hypotony who was treated with 9-0 nylon insertion after PMS implantation.

## Case presentation

**Figure 1:**
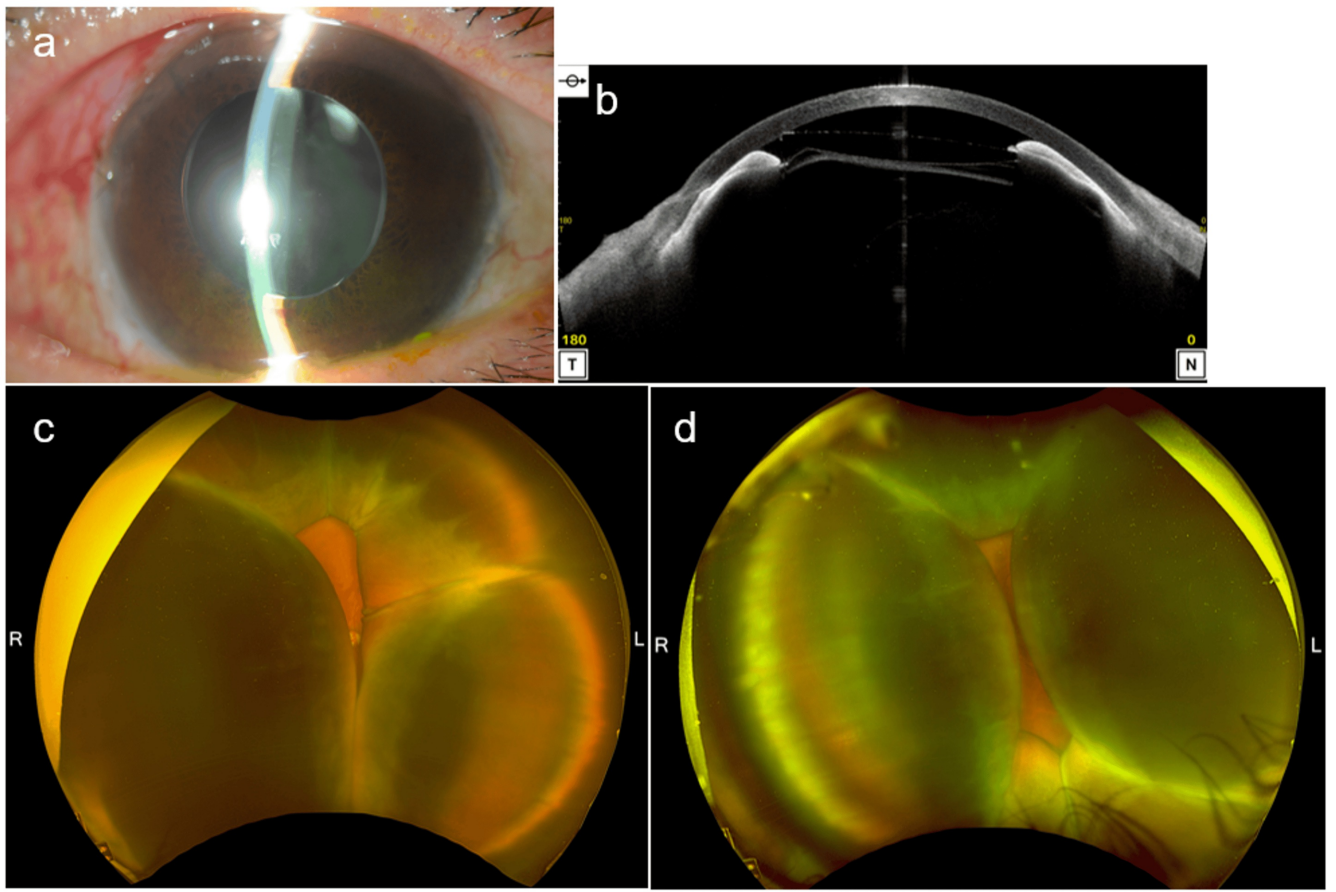
Ocular findings one week after Preserflo MicroShunt surgery. a) Slit-lamp examination revealed a shallow anterior chamber in the right eye. b) Additionally, anterior segment optical coherence tomography (AS-OCT) reveals a shallow anterior chamber and iris contact with the corneal endothelial tissue in the right eye. c,d) Wide-field fundus photographs indicate severe bilateral choroidal detachment nearly touching the opposite retina (c: right eye; d: left eye).

A 78-year-old woman with immunoglobulin G4 (IgG4)-related disease and nephrotic syndrome was being treated with oral steroids for more than 15 years. She presented to the hospital with steroid-induced glaucoma in both eyes. The patient had previously undergone surgery for cataracts in both eyes. At presentation, the ophthalmological examination revealed elevated IOP in both eyes (approaching nearly 40 mmHg) despite the administration of three types of glaucoma eye drops. The patient's visual acuity was 1/10 in the right eye and hand motion in the left eye, which indicated severe visual dysfunction. The fundus was unremarkable, except for enlarged optic disc cupping. Goldmann visual field testing revealed that both eyes had extremely limited residual visual fields. Immediately after the initial examination, we performed PMS implantation surgery on both eyes. The postoperative IOP decreased to 8 mmHg in both eyes the following day; however, we observed CD and anterior chamber collapse in both eyes one week postoperatively (Figure 1). 

**Figure 2:**
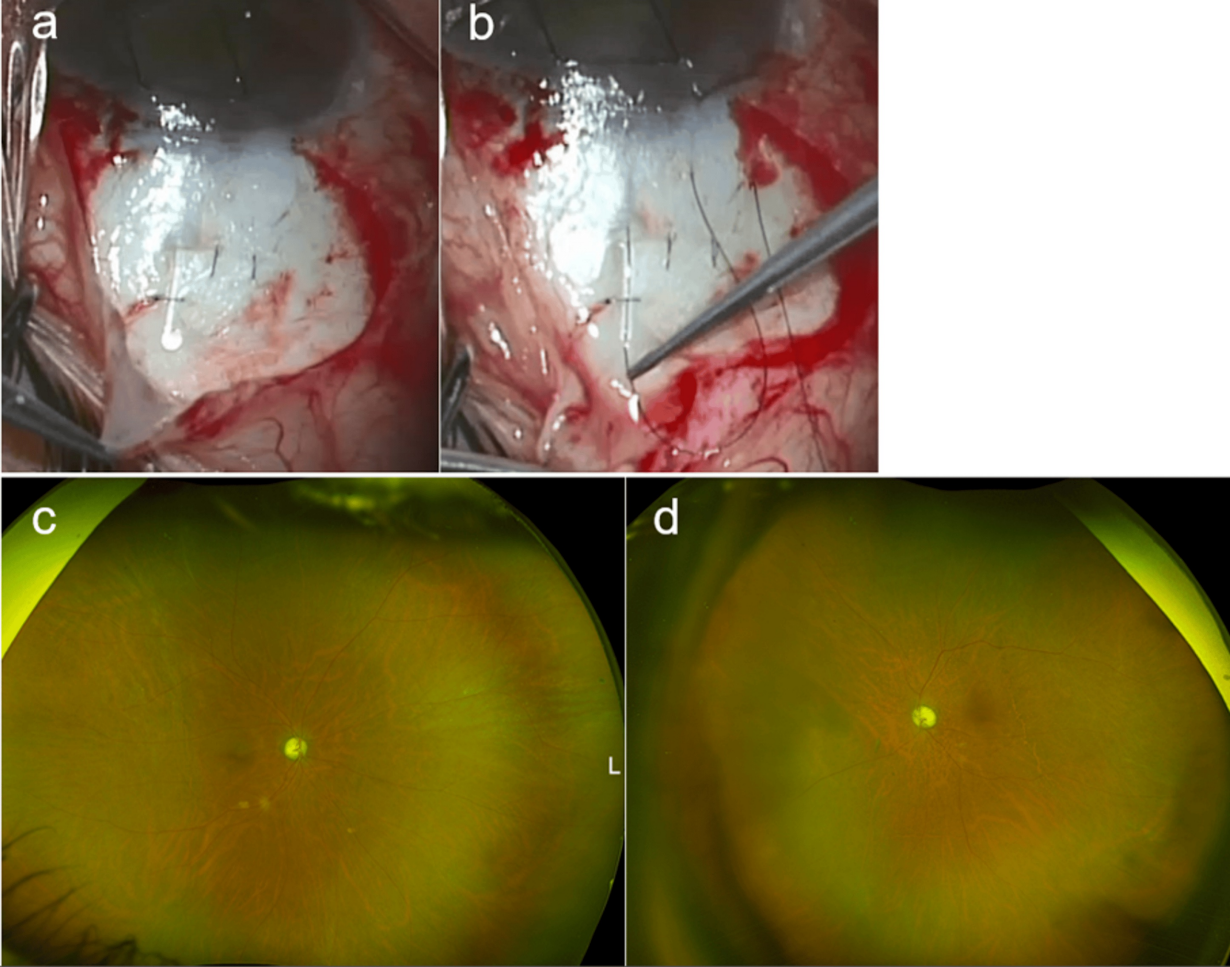
Intraoperative and postoperative findings of intraluminal 9-0 nylon insertion. a) Intraoperative findings before 9-0 nylon insertion. Aqueous humor outflow was observed from the end of the PMS. b) Findings at the time of 9-0 nylon insertion. The 9-0 nylon was inserted into the lumen from the end of the PMS to near the tip using a settling device. The nylon thread was partially exposed near the corneal limbus for ease of removal following surgery. c, d) Wide-field fundus photographs were taken one month after the 9-0 nylon insertion, and choroidal detachment completely resolved in both eyes (c: right eye, d: left eye).

**Figure 3:**
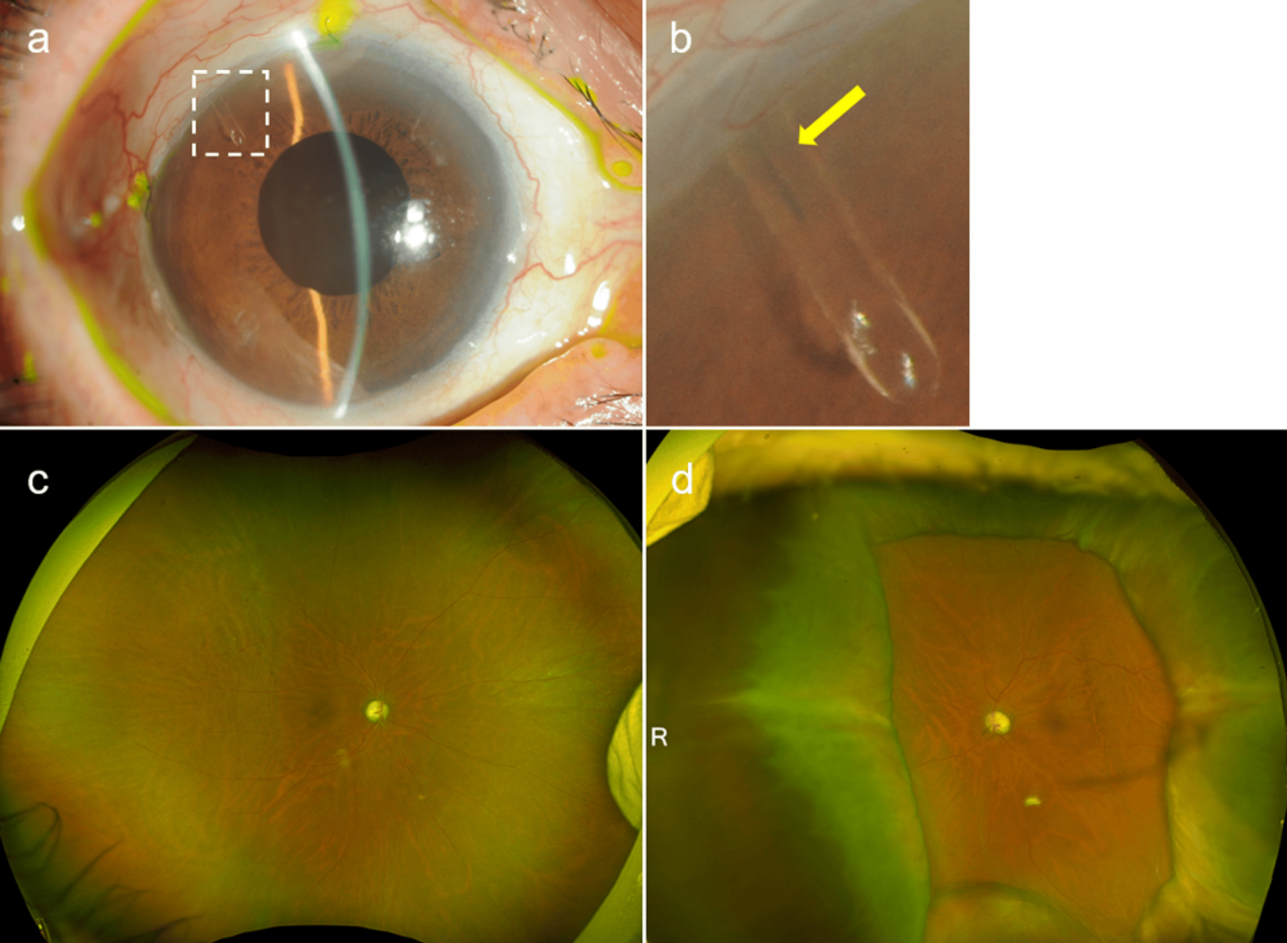
Ocular findings six months after 9-0 nylon implantation. a) Slit-lamp examination of the right eye showed a deep anterior chamber, and the tip of the PMS tube was observed at the superior temporal position. b) Magnificent view of the square part of Figure 1a. The 9-0 nylon remained in situ near the tip of the tube (arrow). c) Fundus findings in the right eye. No recurrence of choroidal detachment was observed. b) Fundus findings in the left eye. Removing the 9-0 nylon one month after the procedure resulted in a recurrence of choroidal detachment.

To elevate the IOP, we injected 1% sodium hyaluronate viscoelastic material into the anterior chamber of both eyes, which increased the IOP to 30 mmHg. However, IOP rapidly decreased, and the CD worsened. We decided to operate with the objective of increasing IOP. Aiming to maintain an elevated IOP, we inserted 9-0 nylon into the lumen of the tube. The suture was advanced into the PMS lumen until it reached the end of the tube. A portion of the suture was anchored to the limbal corneal groove, as described in previous reports [9], to facilitate easy removal later (Figures 2a-2b). After the procedure, the IOP increased from 11 to 28 mmHg. Gradual regression of the CD has been observed since the day after the insertion of the 9-0 nylon stent. Given the postoperative increase in IOP to over 20 mmHg, the insertion of 10-0 nylon in the other eye was considered. However, since CD did not improve at IOP levels in the low 10 mmHg range in this patient, an increase to the upper 10 to 20 mmHg range was deemed desirable. Furthermore, due to the severity of the CD, earlier recovery was prioritized. It was therefore concluded that the insertion of 10-0 nylon might not achieve the IOP necessary for CD regression, and the decision was made to proceed with the insertion of 9-0 nylon in the fellow eye as well. One month after the insertion of the 9-0 nylon, the CD completely resolved in both eyes (Figures 2c-2d). The IOP was 24 mmHg after the administration of a single glaucoma eye drop. The 9-0 nylon inserted into the left eye, the non-dominant eye, was removed. One week after removing the stent in the left eye, its IOP decreased from 24 to 12 mmHg. However, CD recurred in the left eye. Considering that the removal of the stent would result in the recurrence of CD, we decided to retain the stent in the right eye (dominant eye) and continued to follow up with additional glaucoma eye drops. Six months after the insertion of a 9-0 nylon stent, the IOP of the right eye was 14 mmHg with the administration of three types of glaucoma drops and 8 mmHg without glaucoma drops in the left eye. In the right eye, a 9-0 nylon thread was observed within the lumen near the tube tip (Figures 3a-3b). While CD did not recur in the right eye, it persisted in the left eye (Figures 3c-3d). Currently, nine months after the placement of the 9-0 nylon suture, CD remains in the left eye but is gradually diminishing. Fortunately, the patient’s preoperative visual acuity has been preserved.

## Discussion

PMS is a device that can be used to induce low IOP; the Hagen-Poiseuille equation indicates that PMS can be used to induce a theoretical pressure range of 2.6 mmHg, and in vitro experiments have confirmed a similar value [12]. In clinical settings, the scleral tunnel, Tenon's capsule, conjunctival coverings, postoperative inflammation, and tube curvature are considered to contribute to aqueous outflow resistance, which may result in mildly elevated postoperative IOP [13]. Patients with postoperative hypotony are frequently managed with medical therapy, including reduced doses of steroid eye drops to facilitate tissue scarring and atropine eye drops for treating shallow anterior chambers.

Although an optimal surgical treatment remains unestablished, one potential approach involves the injection of viscoelastic materials or air into the anterior chamber. Advantages of this method include technical simplicity and repeatability. However, disadvantages include its transient effect and potential to cause excessive IOP elevation. A recently reported method for resolving this issue involves the implantation of a stent in the tube [9-11]. This approach offers two key advantages: first, it continuously raises the IOP, rather than only temporarily; second, it allows for postoperative IOP adjustment by removal of the stent when the IOP increases. In contrast, the procedure can be considered invasive, and the potential risk of low IOP due to stent removal and the risk of infection associated with long-term stenting remains unclear. An alternative approach is partial ligation of the tube to increase resistance to aqueous humor outflow, as reported in the Ahmed glaucoma valve implantation surgery [14]. However, the tube diameter of the PMS is notably small, achieving proper ligation may therefore be challenging. 

The incidence of low postoperative IOP complications decreased with the insertion of 10-0 nylon [9-11]. Moreover, postoperative improvement of CD is reportedly associated with decreasing IOP by inserting 10-0 nylon into the tube [15]; one report details an ab interno method for inserting 10-0 nylon into the tube without incision of the conjunctiva [16]. In the present case, we initially considered inserting 10-0 nylon. However, after considering the patient's severe visual impairment and the extent of the CD, we instead opted to use 9-0 nylon, which is approximately 10-µm thicker than the 10-0 nylon. We made this decision because our primary objective was to elevate IOP to an adequate level and facilitate rapid recovery from severe CD. We were able to readily insert the 9-0 nylon into the tube; however, upon implantation at the tip, the IOP increased to nearly 30 mmHg, which indicated the necessity for a shorter insertion length. Considering the possibility of recurrent CD after stent removal, as observed in this case, future research should focus on identifying the optimal timing for stent removal.

Elevated preoperative IOP and advanced age have been proposed as risk factors for the development of CD after glaucoma surgery. In the present case, the patient was at a high risk of developing CD owing to her advanced age and markedly elevated preoperative IOP. Additionally, the decreased plasma osmolality resulting from hypoalbuminemia associated with nephrotic syndrome may have contributed to the development of CD by creating an osmotic pressure gradient that facilitates the migration of water from the blood to the interstitium around the choroidal vessels. In patients with such risk factors, aggressive intraoperative nylon stenting is recommended to prevent excessive postoperative IOP decline and reduce the risk of postoperative hypotony.

## Conclusions

To the best of our knowledge, this is the first report detailing the use of 9-0 nylon for postoperative CD following PMS implantation. While the CD improved rapidly, controlling the postoperative IOP was challenging. The insertion of a 9-0 nylon represents a potentially efficacious intervention in specific cases. Nevertheless, the optimization of postoperative IOP and the potential for partial insertion represent a crucial area for future investigation.
